# Identification and diagnosis of mammographic malignant architectural distortion using a deep learning based mask regional convolutional neural network

**DOI:** 10.3389/fonc.2023.1119743

**Published:** 2023-03-22

**Authors:** Yuanyuan Liu, Yunfei Tong, Yun Wan, Ziqiang Xia, Guoyan Yao, Xiaojing Shang, Yan Huang, Lijun Chen, Daniel Q. Chen, Bo Liu

**Affiliations:** ^1^ Department of Radiology, The Second Affiliated Hospital of Guangzhou University of Chinese Medicine, Guangzhou, China; ^2^ Department of Engineering, Shanghai Yanghe Huajian Artificial Intelligence Technology Co., Ltd, Shanghai, China; ^3^ Artificial Intelligence (AI), Research Lab, Boston Meditech Group, Burlington, MA, United States

**Keywords:** deep learning, convolutional neural network, artificial intelligence, malignant architectural distortion, full-field digital mammography

## Abstract

**Background:**

Architectural distortion (AD) is a common imaging manifestation of breast cancer, but is also seen in benign lesions. This study aimed to construct deep learning models using mask regional convolutional neural network (Mask-RCNN) for AD identification in full-field digital mammography (FFDM) and evaluate the performance of models for malignant AD diagnosis.

**Methods:**

This retrospective diagnostic study was conducted at the Second Affiliated Hospital of Guangzhou University of Chinese Medicine between January 2011 and December 2020. Patients with AD in the breast in FFDM were included. Machine learning models for AD identification were developed using the Mask RCNN method. Receiver operating characteristics (ROC) curves, their areas under the curve (AUCs), and recall/sensitivity were used to evaluate the models. Models with the highest AUCs were selected for malignant AD diagnosis.

**Results:**

A total of 349 AD patients (190 with malignant AD) were enrolled. EfficientNetV2, EfficientNetV1, ResNext, and ResNet were developed for AD identification, with AUCs of 0.89, 0.87, 0.81 and 0.79. The AUC of EfficientNetV2 was significantly higher than EfficientNetV1 (0.89 vs. 0.78, P=0.001) for malignant AD diagnosis, and the recall/sensitivity of the EfficientNetV2 model was 0.93.

**Conclusion:**

The Mask-RCNN-based EfficientNetV2 model has a good diagnostic value for malignant AD.

## 1 Introduction

Breast cancer is the most common malignancy in women, ranking the highest incidence among women in the world (1, 2). According to the latest global cancer burden data, the number of new breast cancer cases worldwide reached 2.26 million in 2020, surpassing lung cancer and becoming the most common cancer in the world (1, 2).

Breast cancer screening with mammography is considered effective at reducing breast cancer-related mortality (3, 4). The common imaging manifestations of breast cancer are masses and calcification, followed by architectural distortion (AD). It is sometimes the only manifestation of breast cancer and has important imaging value. On the other hand, AD is seen in malignant and benign lesions (such as sclerosing adenosis, radial scar, postoperative scar, and fat necrosis after trauma, among others). AD is a structural deformation without a defined mass in breast tissue as a fine line or protrusion radiating from a point and a focal contraction, twisted or stiff at the edge of the parenchymal gland (5). AD is more subtle relative to masses and calcification with clear boundaries. AD lesions are often poorly defined, overlap and cover with normal glands, and the lesions are not easy to identify.

There are no established noninvasive standards for the distinction between benign and malignant AD. The literature reported different diagnostic efficacy of various imaging methods for cancer manifesting as AD, such as magnetic resonance imaging (MR) (6), digital breast tomosynthesis (DBT) (7), contrast enhanced spectral mammography (CESM) (8), and the positive predictive value (PPV) varies 34%-88% (8, 9). Amitai et al. reported that the specificity of MRI for AD diagnosis was 68%, and the overall accuracy was 73% (6). Goh et al. reported that the accuracy of CEDM for malignant AD was 72.5% (10). Patel et al. reported that the accuracy of CESM for malignant AD was 82% (8). However the judgment of benign and malignant AD lesions is still difficult for radiologists.

Recently, artificial intelligence (AI) algorithms have been extensively applied in the medical field. Available artificial intelligence (AI) including radiomics and deep learning have been applied to analyze images for detection and diagnosis of lesions in various clinical applications (11–13). Available AI is advanced and approach radiologists’ performance, especially for mammography (7).

The Mask Regional CNN (Mask-RCNN) (14, 15) is a deep neural network aimed at solving instance segmentation problems in machine learning. It effectively combines the two tasks of target detection and image segmentation. However, limited Mask RCNN-based deep learning have been developed for AD identification and malignant AD diagnosis. Rehman et al. recently developed an automated computer-aided diagnostic system using computer vision and deep learning to predict breast cancer based on the architectural distortion on DM and reported great accuracy (16). Xiao et al. proposed two AI methods, radiomics and deep learning, to build diagnostic models for patients presenting with architectural distortion on Digital Breast Tomosynthesis (DBT) images (17).

Although DBT can provide better spatial information for detection and characterization of architectural distortion, many hospital breast X-ray examination are still 2D mammography because of economic reasons not updated equipment timely. Fortunately, AI can be applied to develop fully-automatic computer-aided diagnostic systems (18, 19) and help radiologists improve the diagnostic efficiency (20). Yun et al. underscore the potential of using deep learning methods to enhance the overall accuracy of pretest mammography for malignant AD (20).

Therefore, this study aimed to construct and optimize deep learning models by Mask-RCNN for AD identification in FFDM and evaluate the performance of models for the diagnosis of malignant AD.

## 2 Materials and methods

### 2.1 Study design and participants

This retrospective diagnostic study was conducted at the Second Affiliated Hospital of Guangzhou University of Chinese Medicine between January 2011 and December 2020. Patients with breast AD in full-field digital mammography (FFDM) were included. The inclusion criteria were 1) AD according to the fifth edition of the breast imaging reporting and data system (BI-RADS) diagnostic criteria for architectural distortion (5) and 2) available surgical or biopsy pathological results. The exclusion criteria were 1) patients with obvious mass in the breast or 2) incomplete clinical data or images. Finally, a total of 349 patients were included in this study. Each patient on one side of the breast image contains at least one AD lesions.

### 2.2 Data collection and image annotation

The demographic information and clinical characteristics of the patients were collected, including age, menopause status, pathology, childbirth history, menopausal age, and surgical history. The mammogram images were collected from the Giotto FFDM system (internazionale medico scientifica, IMS, Bologna, Italy). The images for each patient were taken in four standard views: right craniocaudal (R-CC), left craniocaudal (L-CC), right mediolateral oblique (R-MLO), and left mediolateral oblique (L-MLO). The images from the eligible patients were exported to the computer in medical digital imaging and communication (DICOM) format for data anonymization. All images were re-screened to remove substandard images (i.e., incomplete image sequences, poor image quality, artifacts, and cases with clear masses). A total of six radiologists participated in the image processing, and they were divided into three groups, each group including a junior and a senior radiologist. The AD structures on images were outlined and annotated by a group of two experienced radiologists with the ITK-SNAP software (version 3.8). The outlined scope had to include all lesions (such as AD with calcification or asymmetry). The breast fibroglandular tissue (FGT) and imaging features of the lesions including size of lesion, calcification and BI-RADS classification were recorded.

**Figure 1:**
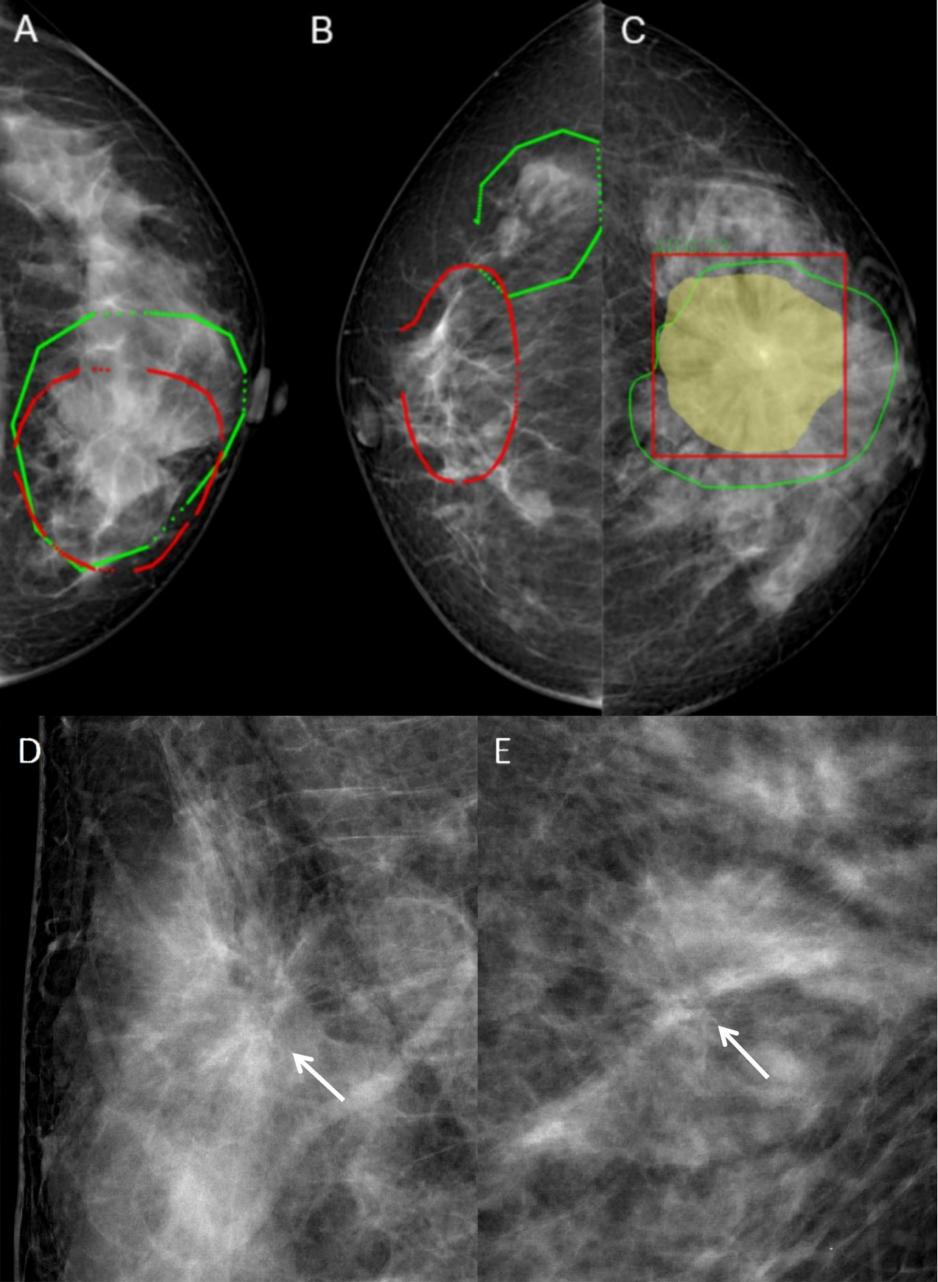
Typical images and image annotation of architectural distortion. **(A)** With intersection over union (IOU) > 0.5, the image was successfully delineated and included in the model training. The red outlined area was marked by the senior radiologist, and the green outlined area was marked by the junior radiologist. **(B)** With IOU <0.5, the image was re-delineated. The red outlined area was marked by the senior radiologist, and the green outlined area was marked by the junior radiologist. **(C)** The green outlined area represents the lesion area delineated by the radiologists, the yellow area represents the machine identification area, and the red box is the lesion range identified by machine learning models. **(D)** FFDM images of a 45-year-old female diagnosed with malignant AD (write arrow). The BI-RADS score is 4C. The pathology was invasive ductal carcinoma. Limited stiffness was seen on the right breast andspiculated margins. **(E)** FFDM images of 64-years-old female with benign AD (write arrow). The BI-RADS score is 4C.The pathology was Complex sclerosinglesion. Stellate shadow and scattered cord were seen on the left breast.


The segmented images were saved in the “.nii” format, and the Python code was used to calculate the regions of interest (ROI) and intersection over union (IOU) to assess the consistency of focal delineation. IOU was calculated according to Equation 1, with A and B representing the ROI area delineated by different radiologists. Images with IOU >0.5 were included in the study (**Figure 1A**), and images with IOU <0.5 were redelineated (**Figure 1B**). All six radiologists participated in the redelineation process, reviewed the film again, and delineated the image again after reaching an agreement (**Figure 1C**). Typical malignant and benign AD images are shown in **Figures 1D, E**.

(1) $$IOU=\frac{A\cap B}{A\cup B}$$

### 2.3 Deep learning model construction

The Mask RCNN was used to construct deep learning models in this study. A combination of training-aware neural architecture search and scaling was used to optimize training speed jointly and to develop these models. The image size of the mammograms used in this study was 2816×3584 pixels. In order to better detect and classify the benign and malignant AD lesions, the images were first scaled to 1024×1024, and Mask RCNN was used to detect and segment the breast AD lesions. There are two stages of Mask RCNN. Both stages are connected to the backbone structure, which is an FPN neural network that consists of a bottom-up pathway, a top-bottom pathway, and lateral connections. The bottom-up pathway can be any ConvNet and Transformer, such as ResNet, ResNext (21, 22), EfficientNetV1 and EfficientNetV2, which extracts the features from raw images. As a network for extracting features, the performance of ResNet and EfficientNet is widely recognized. The ResNet network solves the problem of gradient explosion and training overfitting caused by too deep a network. The ResNext network adds more branches on the basis of ResNet, thereby improving the network’s ability to learn features. EfficientNet V1 and V2 networks use the function of network search on the basis of ResNet, and further enhance the performance and efficiency of the network through parameter combinations. The top-bottom pathway generates a feature pyramid map similar in size to the bottom-up pathway. Lateral connections are convolutions and adding operations between two corresponding levels of the two pathways.

After extracting the ROI of the AD lesions, the ROI was restored to its original image size. A square area containing the lesion area was extracted from the center of the lesion (**Figure 1C**), and the network was used for benign and malignant classification.

### 2.4 Data preprocessing and model training

First, the DICOM data was converted into 16-bit PNG. Then, the image was processed by data augmentation and normalization. Data augmentation includes geometric transformations, color space augmentations, kernel filters, mixing images, and random erasing. Data augmentation can expand samples, prevent overfitting, and improve model robustness. During training, the random erasing method randomly selects a rectangular area in the original image. Then replace the pixels in that area with random values. For the detected and segmented network, the augmented and normalized image and label were scaled to 1024×1024. The ROI of AD lesions is realized by the segmentation algorithm. For the classification of the benign and malignant lesions, lesions often only occupy a small part of the image, so AD lesions are extracted according to the detected ROI, and then the extracted image is scaled to 512×512. Then, 349 patients with 1396 valid images were obtained (349×2×2 = 1396). There were 698 images on one side of the breast with 708 AD lesions in total, with more than one AD lesion detected in some images, and 698 images of contralateral breast without AD lesion. Among all patients, 60% were randomly selected as the training set,20% as the test set, and the other 20% as the validation set. A total of 209 patients with 836 validated images were included in the training set, while 70 patients with 280 images were included in the test set and the same images for the validation set. The training set was used for model training and parameter learning of the model and automatically saved the best model at any time and processed all training data during training. The test set was used for evaluating the diagnostic performance of models. The validation set was applied to save the best model parameters during training and guide the choice of parameters and models.

**Figure 2:**
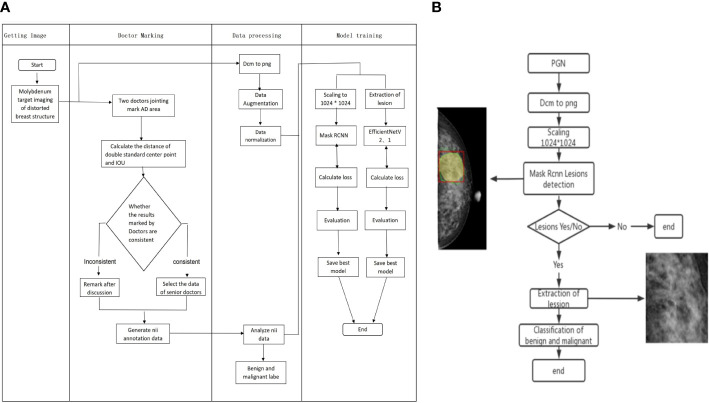
Flow chart of Mask RCNN model training and validation for benign and malignant AD classification. **(A)** The process of Mask RCNN model construction and training. **(B)** The validation of Mask RCNN models for AD classification.

When training the Mask RCNN, four feature extraction networks were selected for comparison: EfficientNetV2, EfficientNetV1, ResNet, and ResNext. During training, Mask RCNN used the multi-task loss, including CrossEntropy Loss for category loss, L1 loss for regression box loss function, and CrossEntropy Loss for the mask loss function. After 50 epochs of training, four network model weights with the best performance in the validation set were obtained. The models with the highest AUC for AD identification were selected as the backbone of Mask RCNN for malignant AD identification. The pathological diagnosis was used as the gold standard for malignant AD diagnosis. The process of Mask RCNN model training and model validation for benign and malignant AD classification is shown in **Figure 2**.

### 2.5 Statistical analysis

Statistical analysis was performed with SPSS 22.0 (IBM, Armonk, NY, USA). The normality test was performed using the Kolmogorov-Smirnov test. Continuous variables with a normal distribution were described as means ± standard deviation (SD) and compared using Student’s t-test. Continuous variables with a skewed distribution were described as median (interquartile range) and compared using Mann-Whitney U-test. Categorical data were presented as n (%) and compared with the chi-square test or Fisher’s exact test. The diagnostic performance of the models was evaluated in the validation set. The receiver operating characteristic (ROC) curve and area under the ROC curve (AUC) were determined, and the Delong test was used for the comparison of AUC. Accuracy, specificity, precision, recall/sensitivity, F1-score, Dice, and Jacc were selected as performance metrics of the deep learning models (**Supplementary Material**). In addition, 95% confidence intervals (CI) were calculated. Two-sided P-values <0.05 were considered statistically significant.

## 3 Results

A total of 349 patients were included: 159 with benign AD and 190 with malignant AD. Patients with malignant AD were aged 49.0 (43.0-56.0) years, and 102 (53.7%) patients were menopausal. Patients with benign AD were aged 48.0 (43.0-52.0) years, and 62 (36.7%) patients were menopausal. There were no differences between the two groups for age (P=0.26), but significantly more patients with malignant AD were menopausal (P=0.01) (**Table 1**). The maximum diameter and vertical diameter of malignant AD are greater than benign AD. The median of maximum diameter and vertical diameter of malignant AD was 1.7cm/1.3cm, the benign AD was 1.1cm/0.9cm. Benign and malignant AD patients had different distributions of FGT (P=0.02) and BI-RADS classification (P<0.001). There were no differences in calcification between the two groups (P=0.23) (**Table 1** **).** In the malignant group, 116 (61.1%) patients had invasive ductal carcinoma (IDC), 12 (6.3%) had invasive lobular carcinoma (ILC), eight (4.2%) had ductal carcinoma *in situ* (DCIS), one (0.5%) had mucinous carcinoma, two had medullary carcinoma (1.1%), 12 had mixed carcinomas (6.3%), and 39 cases were recorded as “breast malignancy” without detailed pathology (the malignant pathology of the 39 cases were further confirmed by a telephone follow-up) (**Table 2**). In the benign group, 92 (57.9%) patients had breast fibrocystic hyperplasia, 17 (10.7%) had postoperative scars, 34 (21.4%) had a radial scar or sclerosing lesion, nine (5.7%) had benign tumors, one (0.6%) had chronic mastitis, and six (3.8%) had breast fibromatosis (**Table 2**).

**Table 1:** Demographic information and image features of AD patients.

Characteristics	Malignant AD (n = 190)	Benign AD (n = 159)	P value
**Age (IQR range), years**	49.0 (43.0-56.0)	48.0 (43.0-52.0)	0.26
**Menopause, n (%)**	102 (53.7%)	62 (36.7%)	0.01
**Size of AD (L/H cm)** **(median)**	1.7/1.3	1.1/0.9	
**FGT**			0.02
**Almost entirely fatty**	1 (0.5%)	2 (1.3%)	
**Scattered densities**	38 (20.0%)	19 (11.9%)	
**Heterogeneous dense**	146 (76.8%)	126 (79.2%)	
**Extremely dense**	5 (2.63%)	12 (7.5%)	
**BI-RADS classification**			<0.001
**II**	6 (3.2%)	10 (6.3%)	
**III**	1 (0.5%)	8 (5.0%)	
**IVa**	3 (1.6%)	15 (9.4%)	
**IVb**	3 (1.6%)	2 (1.3%)	
**IVc**	67 (35.3%)	115 (72.3%)	
**V**	110 (57.9%)	9 (5.7%)	
**Calcification, n (%)**	115 (60.5%)	91 (53.9%)	0.23

AD, architectural distortion; IQR, interquartile range; FGT, fibroglandular tissue. L/H, the median of maximum diameter/maximum vertical diameter of AD.

**Table 2:** Pathology of AD patients with architectural distortion.

Pathology	Cases (n%)
**Malignant AD** **IDC** **ILC**	190 116 (61.1%) 12 (6.3%)
**DCIS**	8 (4.2%)
**mucinous carcinoma**	1 (0.5%)
**medullary carcinoma**	2 (1.1%)
**mixed carcinoma**	12 (6.3%)
**confirmed malignancy without detailed pathology**	39 (20.5%)
**Benign AD**	159
**fibrocystic hyperplasia**	92 (57.9%)
**postoperative scar**	7 (10.7%)
**radial scar or sclerosing lesion**	34 (21.4%)
**benign tumor**	9 (5.7%)
**chronic mastitis**	1 (0.6%)
**breast fibromatosis**	6 (3.8%)

AD, architectural distortion; IDC, invasive ductal carcinoma; ILC, invasive lobular carcinoma; DCIS, ductal carcinoma in situ.

**Figure 3:**
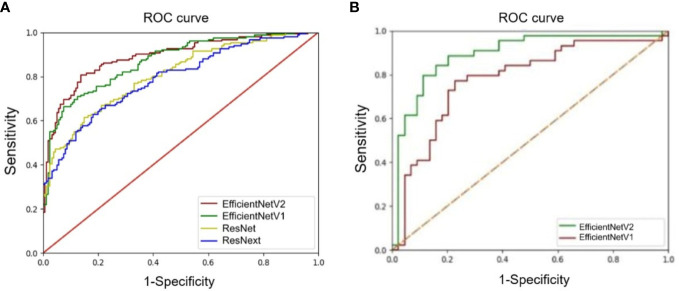
Comparison of receiver operating characteristics (ROC) curves of deep learning models. **(A)** ROC curves of the EfficientNetV1, EfficientNetV2, ResNet, and ResNext models for architectural distortion identification. **(B)** ROC curves of the EfficientNetV1 and EfficientNetV2 models for malignant architectural distortion diagnosis.

The accuracy, precision, recall/sensitivity, F1-score, Dice, Jacc of EfficientNetV2 for AD identification were 0.80, 0.91, 0.66, 0.77, 0.64, and 0.51, while those of EfficientNetV1 were 0.79, 0.84, 0.69, 0.76, 0.63, and 0.50, respectively (**Table 3**). The accuracy of ResNext, and ResNet for AD identification were 0.73 and 0.72, respectively (**Table 3**). The AUCs of EfficientNetV2, EfficientNetV1, ResNext, and ResNet for AD identification were 0.89 (95% CI: 0.83-0.96), 0.87 (95% CI: 0.78-0.93), 0.81 (95% CI: 0.71-0.88), and 0.79 (95% CI: 0.70-0.88), respectively (**Figure 3A**). The EfficientNetV2 model had significantly higher AUC for AD identification than ResNet and ResNext (P=0.005 and P=0.028, respectively), and there was no significant difference in AUC between EfficientNetV2 and EfficientNetV1 in AD identification (P=0.125). Therefore, EfficientNetV2 and EfficientNetV1 were selected as the models for malignant AD diagnosis.

**Table 3:** Diagnostic performances of different models for AD identification.

Validation Set	ResNext	Resnet	EfficientNetV1	EfficientNetV2
**Accuracy**	0.73 (0.62-0.84)	0.72 (0.51-0.85)	0.79 (0.68-0.89)	0.80 (0.72-0.94)
**Precision**	0.74 (0.62-0.84)	0.75 (0.53-0.90)	0.84 (0.80-0.95)	0.91 (0.84-0.98)
**Recall/Sensitivity**	0.68 (0.54-0.80)	0.64 (0.42-0.82)	0.69 (0.56-0.84)	0.66 (0.52-0.74)
**F1-score**	0.71 (0.60-0.80)	0.69 (0.46-0.89)	0.76 (0.66-0.90)	0.77 (0.68-0.90)
**Dice**	0.61 (0.32-0.91)	0.60 (0.27-0.94)	0.63 (0.24-0.90)	0.64 (0.27-0.91)
**Jacc**	0.49 (0.26-0.80)	0.48 (0.24-0.96)	0.50 (0.17-0.82)	0.51 (0.23-0.84)
**AUC**	0.81 (0.71-0.88)	0.79 (0.70-0.88)	0.87 (0.78-0.94)	0.89 (0.83-0.96)
**AUC value differences between models**	0.08 (0.01-0.20) (P=0.028)	0.10 (0.03-0.18) (P=0.005)	0.02 (-0.01-0.10) (P=0.125)	Reference

AD, architectural distortion; AUC, area under the curve.

The AUC of EfficientNetV2 (AUC=0.89, 95% CI: 0.81-0.95) was significantly higher than that of EfficientNetV1 (AUC=0.78, 95% CI: 0.68-0.86) for malignant AD diagnosis (P=0.001) (**Figure 3B**). The accuracy, precision, recall/sensitivity, F1 score, and specificity of EfficientNetV2 for malignant AD diagnosis were 0.84, 0.83, 0.93, 0.84, and 0.74, while those of EfficientNetV1 were 0.82, 0.87, 0.77, 0.82, and 0.87, respectively (**Table 4**).

**Table 4:** The diagnostic performance of EfficientNetV2 and EfficientNetV1 for malignant AD.

Validation set	EfficientNetV2	EfficientNetV1
**Accuracy**	0.84 (0.77-0.95)	0.82 (0.72-0.90)
**Precision**	0.83 (0.72-0.93)	0.87 (0.80-0.96)
**Recall/Sensitivity**	0.93 (0.83-0.98)	0.77 (0.65-0.88)
**F1 score**	0.84 (0.77-0.95)	0.82 (0.72-0.90)
**Specificity**	0.74 (0.62-0.92)	0.87 (0.75-0.95)
**AUC**	0.89 (0.81-0.95)	0.78 (0.68-0.86)
**AUC value differences between models**	0.11 (0.04-0.18) (P=0.001)	Reference

AD, architectural distortion; AUC, area under the curve.

## 4 Discussion

This study constructed four deep learning models based on the Mask RCNN method for AD identification, and the EfficientNetV2 model has a great diagnostic value for malignant AD, with an AUC of 0.89 and recall/sensitivity of 0.93. The EfficientNetV2 model might help radiologists in malignant AD diagnosis, decreasing the need for invasive diagnostic procedures.

Identifying subtle lesions in mammography screening is challenging, with 12.5% of malignancies missed in clinical practice (23, 24). In this study, 110 out of 190 malignant ADs were diagnosed as BI-RADS 5 grade, mainly because the images included typical malignant morphological features or malignant calcification with obvious malignant features. On the other hand, 132 of 159 benign AD were diagnosed with BI-RADS with grade 4, with 115 diagnosed with 4c and nine with grade 5, with a high rate of misdiagnosis as malignant lesions (**Figures 1D, E**). From these two images, both lesions had typical spiculated margins which the radiologists considered malignancy. Because spiculated margins of radiologically detected masses have been well-known morphologic criteria for breast malignancy (25, 26). However, the pathological findings were IDC and sclerosing lesion. It suggested that radiologists could find AD signs in FFDM, but the accuracy of benign vs. malignant differentiation was low, with many cases misdiagnosed as malignant lesions. Therefore, it is of great difficulty for radiologists to identify the AD lesions and differentiate between benign and malignant AD simply based on the morphological characteristics of FFDM observed by the naked eyes.

In routine clinical work, it is necessary to confirm imaging lesions using other imaging methods, such as DBT, Contrast-enhanced mammography (CEM), ultrasound, and MRI, and often an invasive diagnostic method must be performed in case of doubt. It is reported that the negative predictive value of contrast-enhanced MRI (CEMR) is 100% (6), which can help exclude malignant lesions, but CEMR had a low positive predictive value of 30%. It is also reported that the sensitivity, specificity, PPV, and NPV of Contrast-enhanced digital mammography (CEDM) are 100%, 42.6%, 48.5%, and 100% (10) for the diagnosis of malignant AD, respectively. Although the sensitivity of CEMR and CEDM examination is high, they had low positive predictive values, which means that not all the enhanced lesions are malignant, and some are benign lesions. Therefore, even the use of complementary imaging can remain inconclusive. MRI and CEM in routine breast diagnostic tests or screening are not currently standard for AD testing, given the lack of cost-effectiveness and exact diagnostic efficacy (9). A biopsy can provide pathological results, but as an invasive procedure with risks, it brings anxiety to patients and has a chance of a missed diagnosis of malignant foci. Therefore, it is clinically meaningful to find other methods to improve the diagnosis of AD without biopsy.

The fifth edition of the BI-RADS recommends that malignant lesions should be suspected without a definite trauma or surgical history, and further biopsy is recommended (5). Surgical excision has long been advocated for managing DM-detected AD, but more recent evidence suggests that surgical excision of DM-detected AD is not necessary in certain nonmalignant cases, such as when needle biopsy yields radial scar without associated atypia (27–29). Some authors have also proposed that managing nonmalignant architectural distortion on DBT remains controversial; imaging surveillance can be considered for AD on DBT yielding radial scar without atypia or other concordant benign pathologies without atypia at biopsy (30). A biopsy can sometimes provide error pathological results because the needle can sample tissues besides the malignant foci. Therefore, it is clinically meaningful to find other methods to improve the diagnosis of malignant AD without biopsy.

This study aimed to construct an AI model with good diagnostic value for malignant AD. In this study, we built a lesion extraction algorithm corresponding to malignant AD and computed its performance, and at the same time, we used the advanced EfficientNet convolutional neural network with higher computing efficiency and better generalization ability. EfficientNet is based on a lightweight convolutional neural network, which has better model compression ability. It can be widely used in mobile image recognition, object detection, image segmentation, and other tasks, and can meet the more stringent limitations of computing resources (31–34). The EfficientNetV2 model had an accuracy of 0.84, precision of 0.83, recall/sensitivity of 0.93, and F1 score of 0.84 for malignant AD diagnosis, indicating a huge application potential of deep learning models in the diagnosis of malignant AD.

Hand-craft feature extraction techniques showed some value for determining malignant ADs, but these methods still rely on an operator detecting the image features (35). We hope to use deep learning method to establish computer automatic recognition of image features and diagnostic process, to help radiologists improve work efficiency and diagnostic accuracy. Murali et al. used a support vector machine and achieved an accuracy of 90% using 150 AD ROIs (36). Banik et al. examined 4224 ROIs using the gobar filter and phase portrait analysis method and achieved a sensitivity of 90% (37). Jasionowska et al. used a complicated two-step approach (Gobar filter followed by 2-D Fourier transform) and achieved 84% accuracy (38). Other models also achieved relatively good accuracies (39, 40). Still, an issue with deep learning is that the AI model and source of the data can influence the outcomes and that a model that achieves high accuracy with one database might have lower performance with another database. Rehman et al. achieved accuracies of 0.95, 0.97, and 0.98 for the diagnosis of malignant using a larger amount of mammographies from three different databases (16). The reason for their better results may be a database based on larger amounts of data. Fortunately, the increasing availability of mammography databases will help with the development of AI. Even though the present study did not compare multiple imaging methods, the diagnostic accuracy of the EfficientNetV2 model appears promising. It is hoped that AI can be continuously developed and improved in the future, and they may assist radiologists in improving diagnostic accuracy.

This study has some limitations. First, the work is limited by its retrospective design, which leads to some degree of selection bias. Second, only one center was involved, limiting the number of cases. External validation studies are needed. Even with deficiencies, it is still believed that maximizing the clinical application of AI remains an ultimate goal for improving breast cancer screening.

In conclusion, the EfficientNetV2 established based on the Mask RCNN deep learning method has a good diagnostic value for malignant AD. It might help radiologists with malignant AD diagnoses. The results underscore the potential of using deep learning methods to enhance the overall accuracy of mammography for malignant AD.

## Data availability statement

The original contributions presented in the study are included in the article/**Supplementary Material**. Further inquiries can be directed to the corresponding authors.

## Ethics statement

The studies involving human participants were reviewed and approved by the Second Affiliated Hospital of Guangzhou University of Traditional Chinese Medicine (Ethical No. ZE2020-200-01). Written informed consent for participation was not required for this study in accordance with the national legislation and the institutional requirements.

## Author contributions

Conceptualization, BL and DC; Methodology, YL and YT; Software, YT; Validation, YL and YT; Formal Analysis, YT; Investigation, ZX; Resources, GY; Data Curation, GY; Writing – Original Draft Preparation, YL; Writing – Review and Editing, YL; Visualization, YT; Supervision, BL; Project Administration, YW; Funding Acquisition, DC. All authors contributed to the article and approved the submitted version.

## Glossary

**Table:**

AD	architectural distortion
Mask-RCNN	mask regional convolutional neural network
ROC	receiver operating characteristic
AUC	areas under the curve
FFDM	full-field digital mammography
MRI	magnetic resonance imaging
DBT	digital breast tomosynthesis
CESM	contrast enhanced spectral mammography
CEDM	contrast-enhanced digital mammography
CEM	contrast-enhanced mammography
CEMR	contrast-enhanced magnetic resonance imaging
PPV	positive predictive value
NPV	negative predictive value
AI	artificial intelligence
CNN	convolutional neural network
CC	Craniocaudal
MLO	mediolateral oblique
DICOM	digital imaging and communication
FGT	fibroglandular tissue
FPN	Feature Pyramid Networks
BI-RADS	Breast Imaging-Reporting and Data System
ROI	regions of interest
IOU	intersection over union
PNG	Portable Network Graphic
CI	confidence intervals
Dice	dice similarity coefficient
Jacc	Jaccard similarity coefficient
IDC	invasive ductal carcinoma
ILC	invasive lobular carcinoma
DCIS	ductal carcinoma in situ
IQR	Inter-Quartile Range
