# Supplementary material for: Identification and diagnosis of mammographic malignant architectural distortion using a deep learning based mask regional convolutional neural network
Source: Front Oncol. 2023 Mar 22;13:1119743. doi: 10.3389/fonc.2023.1119743 (PMC10075355; doi:10.3389/fonc.2023.1119743)
Supplement: Supplementary file 1 [file DataSheet_1.docx]

**Supplementary material**

The detailed explanation of indicators:

Accuracy = T/(T+N)

Precision = TP/(TP+FP)

Recall = Sensitivity = TP/(TP+FN)

Specificity = TN/(TN+FP)

F1-score = 2*Precision* Recall/ (Precision+ Recall)

Dice = 2*TP/(TP+FP+TP+FN)

Jacc =TP/ (TP+FN+FP)

Abbreviations: T: number of samples correctly classified; N: number of misclassified samples; TP: true positive; FP: false positive; TN: true negative; FN: false negative
